# Who Really Benefits from CPAP? Disease Severity, Response Quality, and Long-Term Survival in Obstructive Sleep Apnea

**DOI:** 10.3390/arm94040055

**Published:** 2026-07-28

**Authors:** Wojciech Kuczyński, Karol Pierzchała, Weronika Bielska, Zuzanna Boczar, Aleksandra Kudrycka, Piotr Białasiewicz

**Affiliations:** Department of Sleep Medicine and Metabolic Disorders, Medical University of Lodz, 6/8 Mazowiecka Street, 92-215 Łódź, Poland; karol.pierzchala@stud.umed.lodz.pl (K.P.); weronika.bielska@stud.umed.lodz.pl (W.B.); zuzanna.boczar@stud.umed.lodz.pl (Z.B.); aleksandra.kudrycka@umed.lodz.pl (A.K.); piotr.bialasiewicz@umed.lodz.pl (P.B.)

**Keywords:** obstructive sleep apnea, CPAP, mortality, nocturnal hypoxaemia, competing risks, precision medicine, respiratory medicine

## Abstract

**Highlights:**

**What are the main findings?**

- The survival benefit of CPAP in obstructive sleep apnea is not uniform—it is concentrated in severe disease and graded by the quality of the therapeutic response (residual AHI on home CPAP).
- Nocturnal hypoxaemic burden (time with SpO_2_ < 90%), rather than CPAP itself, is the dominant independent driver of pulmonary mortality.

**What are the implications of the main findings?**

- CPAP resources and adherence support should be prioritised for patients with severe OSA, with device-recorded residual AHI monitored as a modifiable treatment target.
- Oximetric endpoints deserve explicit attention alongside the AHI when titrating therapy and designing trials, enabling severity- and response-stratified respiratory care.

**Abstract:**

Obstructive sleep apnea (OSA) is associated with increased cardiovascular, respiratory, and all-cause mortality, yet the long-term survival impact of continuous positive airway pressure (CPAP) remains contested, and treatment is usually analysed as a binary exposure rather than by the quality of the response achieved. In a single-centre cohort of 4368 adults referred for polysomnography and followed for 8–20 years (prespecified subgroup with apnea–hypopnea index [AHI] ≥ 15, n = 2304), we applied cause-specific Cox and Fine–Gray competing-risks models, together with machine-learning classifiers, to characterise all-cause, cardiovascular, and pulmonary mortality. CPAP was associated with reduced all-cause (hazard ratio [HR] 0.75), cardiovascular (HR 0.75), and pulmonary mortality (HR 0.54), with the benefit confined to severe OSA (HR 0.66) and absent in moderate disease (HR 0.95). Good responders showed a significant 34% reduction in all-cause mortality (HR 0.66), whereas poor responders showed no significant reduction. Nocturnal desaturation (time with oxygen saturation < 90%) was the dominant independent predictor of pulmonary mortality, and baseline features predicted response category only moderately (macro-averaged AUC 0.76). Long-term CPAP confers a severity- and response-dependent survival benefit; nocturnal hypoxaemia is a key, modifiable driver of respiratory death.

## 1. Introduction

Obstructive sleep apnea (OSA) is a highly prevalent chronic respiratory disorder characterised by repetitive episodes of partial or complete upper airway obstruction during sleep, resulting in intermittent hypoxia, sleep fragmentation, and sustained activation of the sympathetic nervous system. Beyond its well-established cardiovascular consequences—hypertension, cardiac arrhythmias, coronary artery disease, stroke, and heart failure—OSA imposes a substantial and frequently underappreciated burden on the respiratory system through chronic intermittent hypoxaemia. A meta-analysis of six prospective observational studies encompassing nearly 12,000 patients reported that severe OSA was associated with a pooled hazard ratio of 1.90 (95% CI 1.29–2.81) for all-cause mortality and 2.65 (95% CI 1.82–3.85) for cardiovascular mortality [1]. Whether effective treatment translates into a meaningful reduction in mortality remains one of the most clinically consequential and contested issues in respiratory and sleep medicine.

Continuous positive airway pressure (CPAP) remains the cornerstone of OSA therapy. Beyond reducing the apnea–hypopnea index (AHI) and alleviating daytime sleepiness, CPAP attenuates nocturnal blood pressure surges, reduces sympathetic overdrive, improves endothelial function, and mitigates systemic inflammation [2,3]. Observational evidence has supported a mortality benefit: untreated severe OSA was independently associated with cardiovascular death in elderly patients, a risk significantly reduced by adequate CPAP treatment [4]. Landmark data in men confirmed higher rates of fatal and non-fatal cardiovascular events in untreated compared with CPAP-treated patients [5], a pattern subsequently confirmed in female cohorts [6].

The landscape shifted with large-scale randomised controlled trials. The SAVE trial, enrolling 2717 adults with moderate-to-severe OSA and established cardiovascular disease, found no significant reduction in the primary composite cardiovascular endpoint (HR 1.10, 95% CI 0.91–1.32) [7]. A meta-analysis found CPAP associated with reduced events in observational studies (RR 0.61, 95% CI 0.39–0.94), but not in RCTs (RR 0.57, 95% CI 0.32–1.02) [8]. This discrepancy has been attributed to the “healthy user” effect in observational cohorts, and insufficient CPAP adherence in pragmatic trials [7,8].

Treatment adherence appears critical: protective effects are consistently observed at ≥4 h per night, with a dose–response extending to ≥8 h [9,10,11]. Benefits may also be delayed, becoming apparent only after years of sustained treatment [12,13], and studies enrolling patients at highest cardiovascular risk have demonstrated the largest absolute mortality differences [4,14,15]. A recent meta-analysis of over one million patients has sought to reconcile these discordant findings [16]. Whether the null RCT findings reflect a true absence of benefit or are a consequence of insufficient adherence, suboptimal patient selection, and inadequate follow-up remains unresolved.

A critical limitation common to nearly all prior work is the treatment of CPAP as a binary exposure (treated vs. untreated), which ignores the wide variation in the physiological response actually achieved during real-world home use. Two patients prescribed CPAP may differ substantially in residual disease burden and residual nocturnal hypoxaemia, yet conventional analyses assign them identical exposure status. We hypothesised that the quality of the therapeutic response—quantified objectively from device-recorded residual AHI across home therapy nights—would carry prognostic information beyond CPAP prescription alone, and that the survival benefit of CPAP would be concentrated in patients with more severe disease. In a single-centre cohort of 4368 adults followed for up to 20 years with complete national death-registry linkage, we therefore (i) quantified the association of CPAP therapy with all-cause, cardiovascular, and pulmonary mortality across the spectrum of OSA severity; (ii) introduced and evaluated a response-quality classification based on residual AHI; and (iii) used complementary survival models (cause-specific Cox, Fine–Gray competing risks) and machine learning to disentangle the prognostic roles of residual AHI control and nocturnal hypoxaemic burden, and, ultimately, to identify which patients are the true beneficiaries of CPAP therapy.

## 2. Materials and Methods

### 2.1. Study Population and Data Source

We analysed a single-centre cohort of 4368 consecutive patients referred for full-night attended polysomnography at the Department of Sleep Medicine and Metabolic Disorders, Medical University of Lodz, Poland. Cox proportional hazards regression and Random Survival Forest (RSF) mortality models were applied to both the full cohort (n = 4368) and a prespecified subgroup of patients with an apnea–hypopnea index ≥ 15 events/hour (n = 2304). Participants were followed for 8 to 20 years, during which 1017 individuals died and 3351 (76.72%) remained alive at the end of follow-up (Table 1). In the AHI ≥ 15 subgroup, 737 deaths occurred (32.0%). Exclusion criteria included age < 18 years and BMI < 18 kg/m^2^.

OSA severity was classified as normal (AHI < 5), mild (AHI 5–14.9), moderate (AHI 15–29.9), or severe (AHI ≥ 30). All-cause mortality was ascertained through linkage with national registry data; cause of death was coded using ICD-10 and grouped by organ system (Table 2).

Among the 1017 deceased patients (23.28%), cardiovascular diseases were the leading cause of death (n = 335; 7.67% of the total cohort), followed by neoplasms (n = 243; 5.56%) and respiratory diseases (n = 101; 2.31%) (Table 2).

### 2.2. CPAP Therapy

CPAP therapy was defined as ≥4 h per night on at least 75% of nights. Under this definition, 956 patients (41.49%) in the AHI ≥ 15 subgroup were classified as CPAP-treated. The median duration of CPAP therapy was 169 days (IQR 58–683; range 15–4708 days).

The median CPAP therapy duration of 169 days (IQR 58–683) refers to the period over which device-recorded residual-AHI data were available for each treated patient—the window used to characterise response quality—and does not represent the total lifetime duration of CPAP use. In the survival models, CPAP was modelled as a fixed baseline (ever-treated) exposure rather than as a time-varying covariate.

CPAP was offered to all patients meeting clinical criteria; the specific reason a given patient did not ultimately receive it (non-acceptance, intolerance, or clinical unsuitability) was not systematically recorded, so the No-CPAP group is heterogeneous and a degree of treatment-selection bias cannot be excluded.

### 2.3. Cox Proportional Hazards Regression

Cox proportional hazards (PH) regression was performed separately in the AHI ≥ 15 subgroup and the full cohort. Three multivariable models were fitted: (i) a binary CPAP therapy model (yes/no), (ii) a model incorporating a multiplicative AHI × CPAP interaction term to assess severity-dependent treatment effects, and (iii) a CPAP response classification model replacing the binary CPAP variable with three-category response (Good, Poor, No CPAP). All multivariable models were adjusted for age, sex, BMI, AHI, oxygen desaturation (Des ≤ 90% TST), smoking, hypertension, diabetes mellitus, and hypothyroidism. The proportional hazards assumption was verified using Schoenfeld residuals. Discrimination was assessed by the concordance index (C-index) and calibration by the Integrated Brier Score (IBS).

### 2.4. Cause-Specific Cox Regression

Cause-specific Cox proportional hazards models were fitted separately for cardiovascular and pulmonary mortality. In each model, deaths from all other causes were treated as censored observations at the time of their occurrence. This approach estimates the instantaneous hazard of the event of interest among individuals who have not yet experienced any event. Univariable models evaluated each clinical, demographic, and polysomnographic variable individually. Multivariable models were adjusted for age, sex, BMI, AHI, oxygen desaturation (Des ≤ 90% TST), smoking, hypertension, diabetes mellitus, and hypothyroidism. Separate multivariable models were fitted for binary CPAP therapy and CPAP response classification.

### 2.5. Fine–Gray Competing Risks Regression

To complement the cause-specific Cox models, Fine–Gray subdistribution hazard regression was performed for cardiovascular and pulmonary mortality. Unlike cause-specific hazards, the Fine–Gray model directly quantifies the effect of covariates on the cumulative incidence function (CIF) while retaining subjects who die of competing causes in the risk set. This is particularly relevant here, as non-cardiovascular and non-pulmonary deaths constitute a substantial proportion of events. Univariable models assessed each sleep-related and treatment variable individually; multivariable models were adjusted for age, sex, BMI, smoking, hypertension, diabetes, and dyslipidemia.

### 2.6. CPAP AHI Measurement and Response Classification

The residual AHI on CPAP (AHI_CPAP_) was calculated as the mean AHI recorded by the CPAP device, averaged across all therapy nights on which the patient actively used the device at home. The AHI reduction was computed as [AHI_diagnostic_ − AHI_CPAP_]/AHI_diagnostic_ × 100. Patients were classified into response categories as follows. Good response: AHI reduction ≥ 50% and residual AHI < 10 events/h. Poor response: AHI reduction < 50%, or AHI reduction ≥ 50%, but residual AHI ≥ 10. No CPAP: did not receive CPAP therapy (reference category).

The response thresholds were selected to reflect current clinical practice for defining adequate CPAP control. A residual AHI below 10 events/hour corresponds to the widely used target for effective therapy, whereas the additional requirement of at least a 50% reduction from the diagnostic AHI ensures that classification as a good responder reflects a substantial improvement relative to each patient’s own baseline rather than a low residual value arising merely from a modest starting AHI. Patients meeting only one of these two criteria were classified as poor responders. These cut-offs are pragmatic and, to our knowledge, not yet formally standardised; they are intended as a reproducible, clinically interpretable operationalisation of response quality and warrant external validation.

A Random Forest classifier (500 trees) and a regularised multinomial logistic regression were trained to predict CPAP response category (Good, Poor, No CPAP) using stratified cross-validation. Performance was summarised with accuracy, macro F1, and macro-averaged AUC.

### 2.7. Statistical Analysis

Spearman rank correlations were used for continuous variables; Mann–Whitney U and Kruskal–Wallis tests for categorical comparisons; post hoc pairwise tests used Dunn’s method with Bonferroni correction. Analyses were performed in Python (version 3.14) (lifelines, scikit-survival, scikit-learn). A two-sided *p* < 0.05 was considered statistically significant.

This study was conducted in compliance with the amended Declaration of Helsinki, and the Bioethics Committee of the Medical University of Łódź, Poland, approved the study protocol (RNN/23/15/KE; RNN/393/19/KE). This study was neither funded by an institutional grant nor by the pharmaceutical industry or a medical company.

## 3. Results

### 3.1. Proportional Hazards Regression

Cox regression results across three multivariable models are presented in Table 3. In the binary CPAP therapy model, CPAP was independently associated with significantly reduced all-cause mortality in both the AHI ≥ 15 subgroup (0.75 [0.65–0.88]; *p* < 0.001) and the full cohort (0.82 [0.71–0.94]; *p* = 0.006), corresponding to 25% and 18% reductions, respectively. For cardiovascular mortality, the benefit reached significance in the AHI ≥ 15 subgroup (0.75 [0.58–0.97]; *p* < 0.05), but not the full cohort. Pulmonary mortality was significantly reduced in both populations (AHI ≥ 15: 0.54 [0.33–0.90]; full cohort: 0.56 [0.35–0.89]; both *p* < 0.05).

In the interaction model, the AHI × CPAP interaction confirmed that the survival hazard attributable to OSA severity was substantially attenuated in CPAP-treated patients. When CPAP was stratified by response quality, good responders showed a significantly reduced all-cause mortality in both the AHI ≥ 15 subgroup (0.66 [0.56–0.78], *p* < 0.001) and the full cohort (0.76 [0.65–0.88], *p* < 0.001), whereas the reduction in poor responders did not reach statistical significance (AHI ≥ 15: 0.83 [0.68–1.01], *p* = 0.066; full cohort: 0.93 [0.77–1.12], *p* = 0.451). For pulmonary mortality, good response was significantly associated with reduced risk in both the AHI ≥ 15 subgroup (0.58 [0.34–0.99]; *p* = 0.044) and the full cohort (0.60 [0.37–0.99]; *p* = 0.048), whereas poor response showed no significant association. The multivariable model achieved a C-index of 0.764 in the full cohort and 0.731 (IBS = 0.147) in the subgroup. Corresponding univariable cause-specific hazard ratios for cardiovascular and pulmonary mortality are presented in Table 4, where higher AHI and greater nocturnal desaturation were each individually associated with both cardiovascular and pulmonary mortality.

### 3.2. Severity- and Response-Stratified Survival

**Figure 1:**
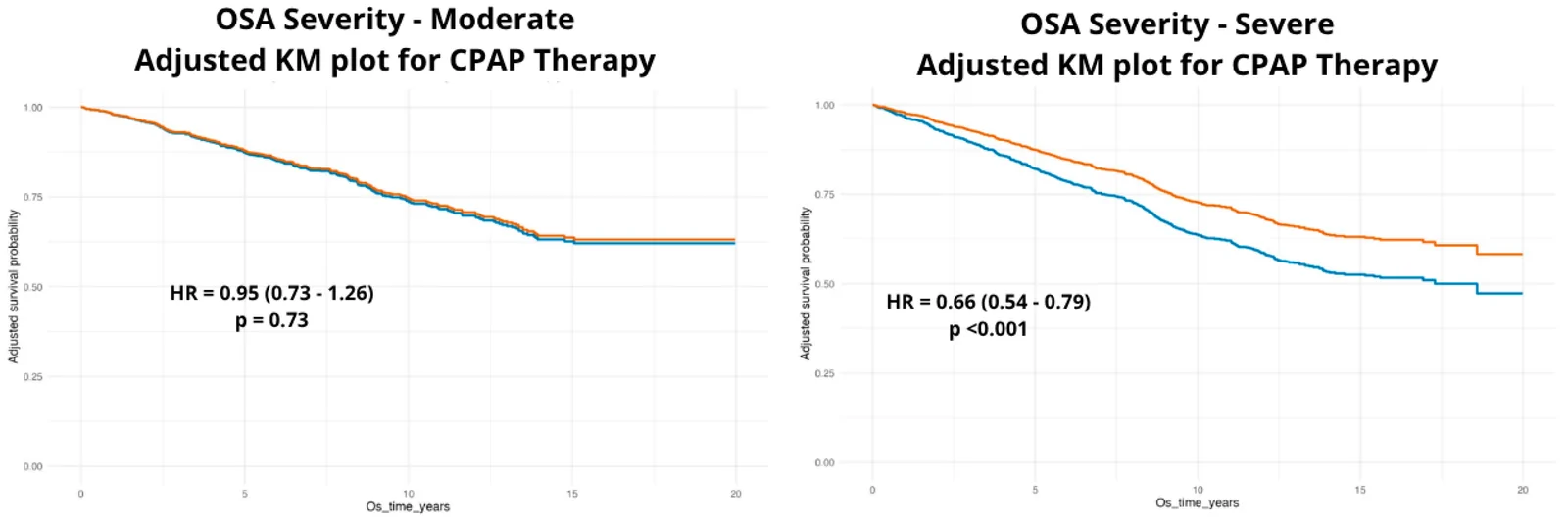
Adjusted Kaplan–Meier plot by OSA severity. Left panel: moderate OSA (AHI 15–29.9), HR 0.95 (0.73–1.26), *p* = 0.73. Right panel: severe OSA (AHI ≥ 30), HR 0.66 (0.54–0.79), *p* < 0.001. The survival benefit of CPAP therapy was significant only in the severe OSA subgroup. Red—CPAP good response, blue—no CPAP.

Adjusted Kaplan–Meier survival curves stratified by OSA severity (Figure 1) demonstrated a significant all-cause mortality reduction with CPAP therapy in the severe OSA subgroup (HR 0.66, 95% CI 0.54–0.79, *p* < 0.001), whereas no significant benefit was observed in moderate OSA (HR 0.95, 95% CI 0.73–1.26, *p* = 0.73).

**Figure 2:**
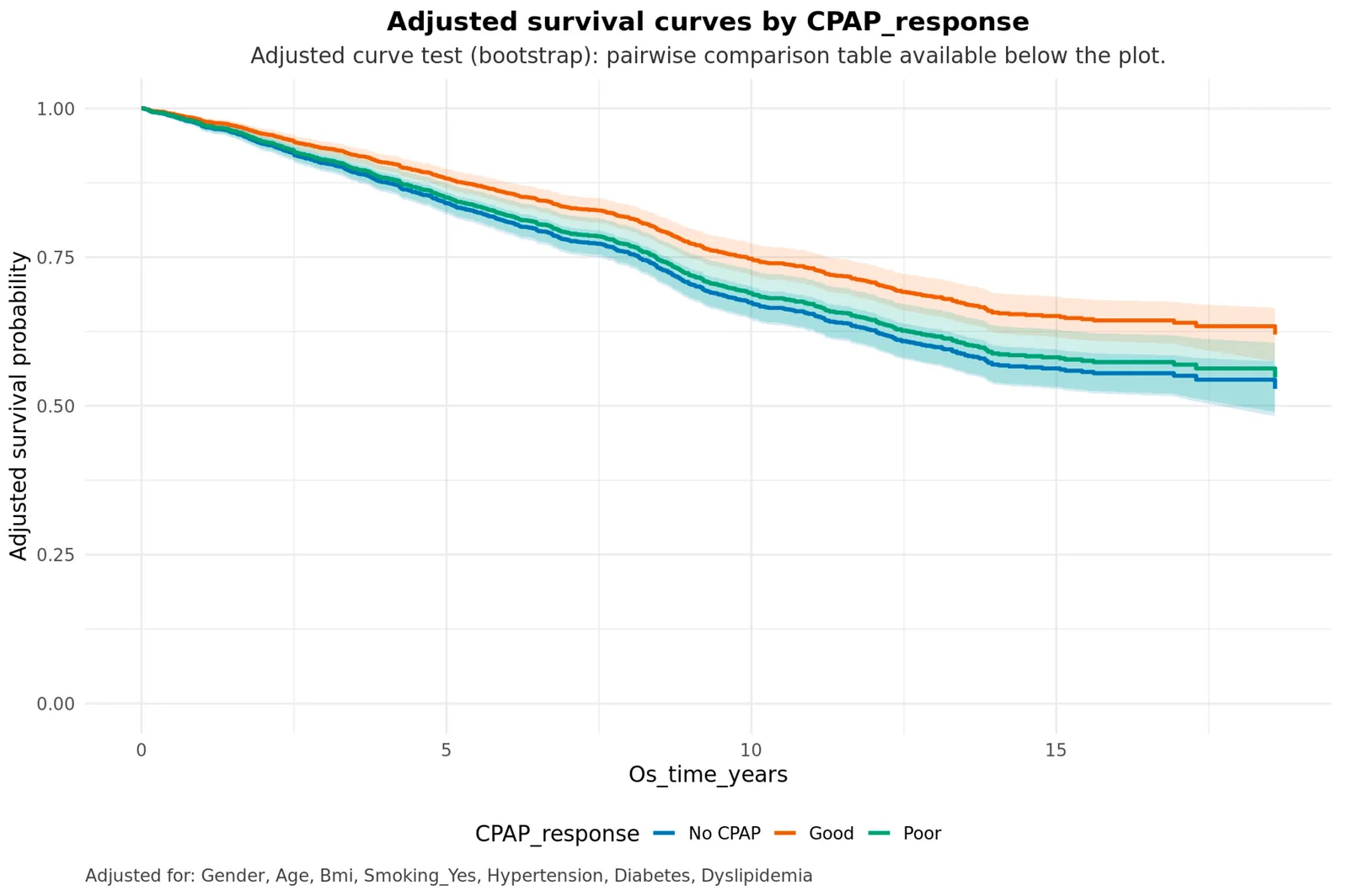
Adjusted survival curves by CPAP response category in the AHI ≥ 15 subgroup (n = 2304): Good response (n = 845), Poor response (n = 412), and No CPAP (n = 1047, reference); curves adjusted for sex, age, BMI, smoking, hypertension, diabetes, and dyslipidemia. Pairwise adjusted-curve test (area between curves; 500 bootstrap resamples): No CPAP vs. Good *p* < 0.001; No CPAP vs. Poor *p* = 0.510; Good vs. Poor *p* = 0.005.

Adjusted survival curves stratified by CPAP response category in the AHI ≥ 15 subgroup (Figure 2) showed significantly higher survival in good responders than in both untreated patients (adjusted-curve bootstrap test, *p* < 0.001) and poor responders (*p* = 0.005), whereas poor responders did not differ significantly from the No-CPAP group (*p* = 0.510).

### 3.3. Fine–Gray Competing Risks Analysis

**Figure 3:**
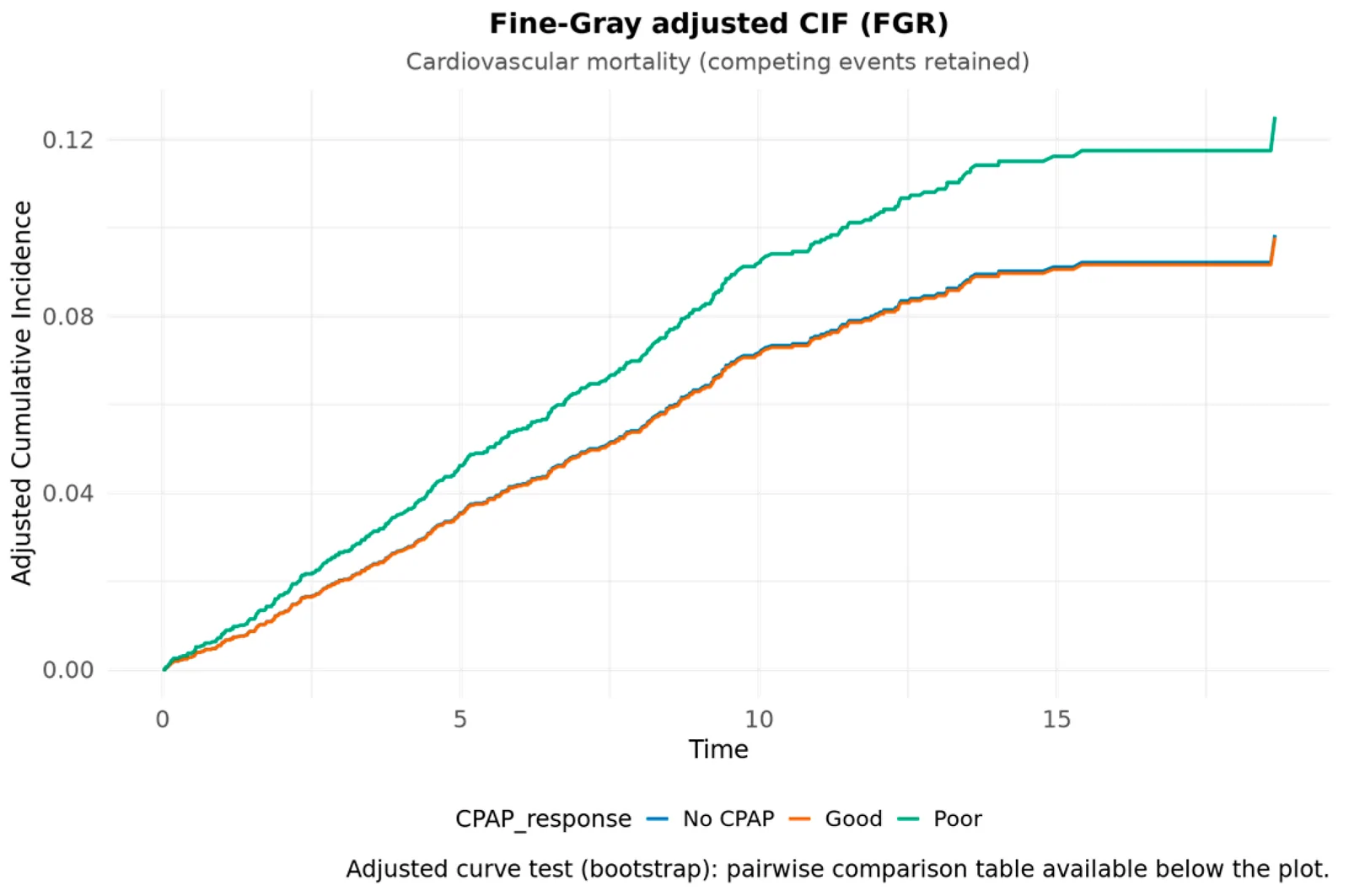
Adjusted cumulative incidence function (CIF) for cardiovascular mortality (Fine–Gray) by CPAP response category in the AHI ≥ 15 subgroup: No CPAP (n = 1047, blue), poor (n = 412, green), and good responders (n = 845, orange); adjusted for sex, age, BMI, smoking, hypertension, diabetes, and dyslipidemia. Pairwise adjusted-curve test (area between curves; N = 500 bootstrap resamples): No CPAP vs. Good *p* = 0.955; No CPAP vs. Poor *p* = 0.080; Good vs. Poor *p* = 0.080. No significant differences in cardiovascular cumulative incidence were observed across CPAP response groups.

**Figure 4:**
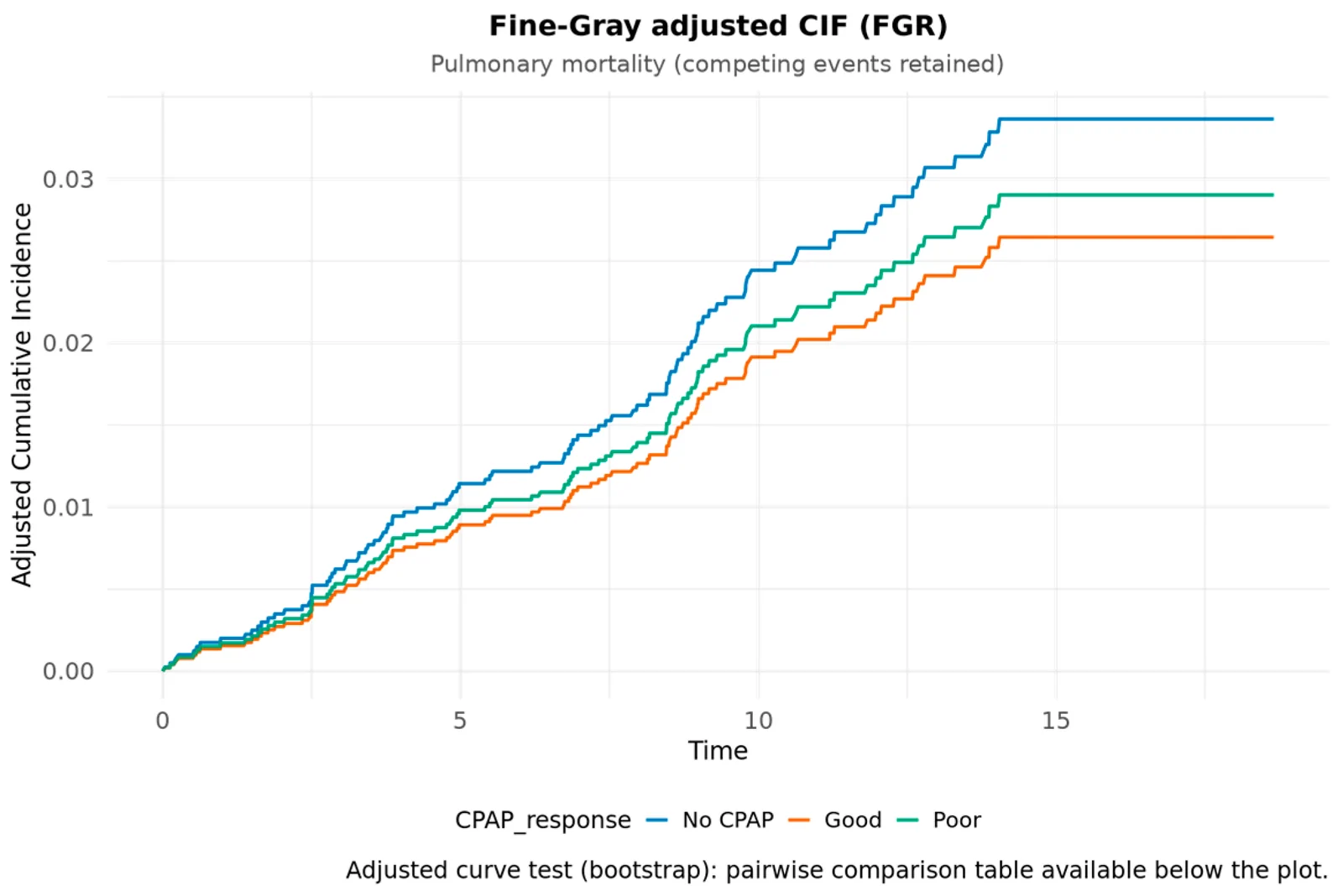
Adjusted cumulative incidence function (CIF) for pulmonary mortality (Fine–Gray) by CPAP response category in the AHI ≥ 15 subgroup: No CPAP (n = 1047, blue), poor (n = 412, green), and good responders (n = 845, orange); adjusted for sex, age, BMI, smoking, hypertension, diabetes, and dyslipidemia. Pairwise adjusted-curve test (area between curves; N = 500 bootstrap resamples): No CPAP vs. Good *p* = 0.265; No CPAP vs. Poor *p* = 0.605; Good vs. Poor *p* = 0.810. No significant differences were observed, although good responders showed the numerically lowest pulmonary cumulative incidence.

Fine–Gray subdistribution hazard models were fitted for cardiovascular and pulmonary mortality to account for the competing risk of death from other causes, and the adjusted cumulative incidence functions were compared across CPAP response categories using a non-parametric bootstrap test on the area between curves (N = 500 resamples). For cardiovascular mortality (Figure 3), no significant differences in adjusted cumulative incidence were observed between groups (No CPAP vs. Good *p* = 0.955; No CPAP vs. Poor *p* = 0.080; Good vs. Poor *p* = 0.080), with poor responders showing the numerically highest incidence. For pulmonary mortality (Figure 4), the differences were likewise non-significant (No CPAP vs. Good *p* = 0.265; No CPAP vs. Poor *p* = 0.605; Good vs. Poor *p* = 0.810), although good responders showed the numerically lowest cumulative incidence.

### 3.4. Prediction of CPAP Response

**Figure 5:**
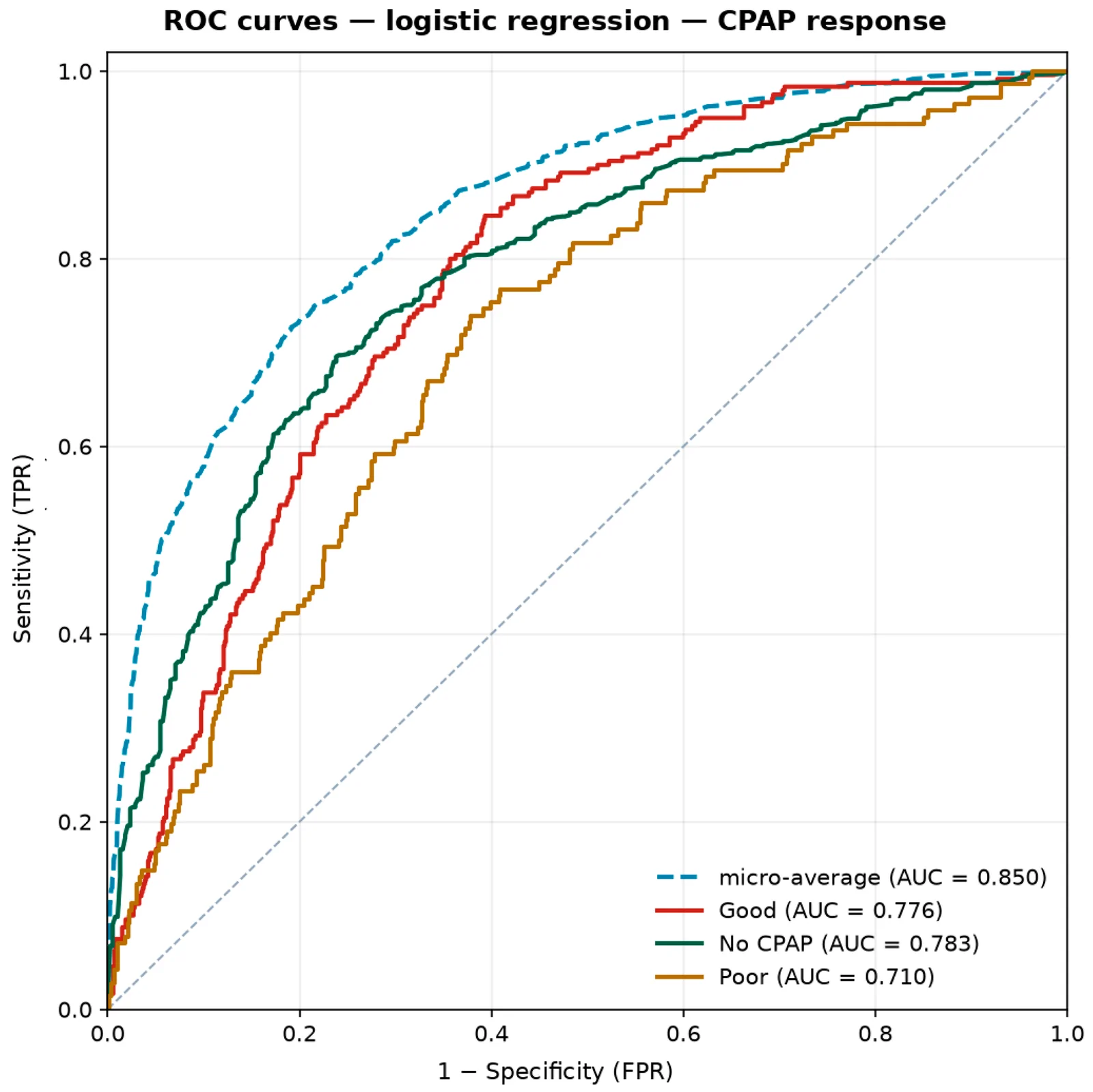
Receiver operating characteristic (ROC) curves for the logistic-regression classifier predicting CPAP response category. Per-class one-versus-rest AUC: No CPAP 0.783, good responders 0.776, poor responders 0.710; macro-averaged AUC 0.756 (micro-average 0.850). Because macro-averaged metrics can be inflated by class imbalance, the confusion matrix is additionally provided in Supplementary Figure S1.

A supervised classifier (multinomial logistic regression) was trained to predict CPAP response category from baseline clinical and polysomnographic features and evaluated on a held-out test set. Discrimination was moderate: overall accuracy 68.1%, balanced accuracy 47.9%, macro-averaged one-versus-rest AUC 0.756 (per-class: No CPAP 0.783, good responders 0.776, poor responders 0.710), and macro F1 0.49, see Figure 5. Because macro-averaged metrics can be inflated by the dominant No-CPAP class, performance was additionally examined with a confusion matrix and per-class metrics (Supplementary Figure S1): the model identified No-CPAP patients well (recall 88%), but discriminated the minority good- and poor-responder classes only modestly (recall 43% and 13%, respectively). OSA severity was by far the most informative baseline feature, followed by statin use and BMI. These analyses are therefore exploratory and hypothesis-generating rather than a deployable predictive tool.

The machine-learning analyses are exploratory and hypothesis-generating rather than a deployable tool. Their intended value is clinical direction: because CPAP response category can be predicted from routinely available baseline polysomnographic and clinical features, these models could form the basis of a future decision-support algorithm that indicates, already at the point of diagnosis, which patients are most likely to respond well to CPAP and therefore benefit from it, thereby helping clinicians prioritise therapy and enrich the design of future randomised trials. The survival analyses remain the central contribution of this work.

### 3.5. Associations Between CPAP Response and Baseline Variables

CPAP response category was significantly associated with a broad range of polysomnographic, demographic, and clinical variables (Table 5 and Table 6). Good and poor responders had significantly higher baseline AHI, obstructive event counts, and nocturnal desaturation than the no-CPAP group. Age was comparable between poor and good responders; BMI was elevated in both CPAP-treated groups relative to no CPAP. Male sex, hypertension, diabetes, dyslipidemia, and myocardial infarction were significantly more prevalent in the poor-response group (all *p* < 0.05). Higher rates of statins, hypoglycaemic drugs, insulin, and acetylsalicylic acid among poor responders confirmed that this group represents a more comorbid, metabolically burdened phenotype. Daytime sleepiness was assessed using the Epworth Sleepiness Scale (ESS; range 0–24, >10 indicating excessive daytime sleepiness); the Back AHI index refers to the apnea–hypopnea index recorded specifically during the supine sleeping position.

The on-treatment residual AHI (AHI_CPAP) and the percentage reduction from baseline are now reported for each response category in Table 5, confirming that classification is based on post-treatment values: good responders achieved a markedly lower residual AHI and a larger percentage reduction than poor responders, while their baseline AHI was comparable between the two groups. The No-CPAP group has no AHI_CPAP by definition.

## 4. Discussion

This study demonstrates that CPAP therapy confers a significant, severity-dependent survival benefit in patients with moderate-to-severe OSA, with the magnitude of benefit further determined by the quality of the therapeutic response. The central message is therefore one of patient selection: the true beneficiaries of CPAP are patients with severe disease who achieve effective control of residual events and nocturnal hypoxaemia during home use, rather than the OSA population at large. The AHI × CPAP interaction and stratified survival analyses converge on a consistent finding: the mortality benefit of CPAP is greatest in more severe disease. This severity-dependent pattern is consistent with landmark observational studies [4,5,6] and with the biological plausibility of treating a condition whose pathophysiological sequelae scale with event frequency and hypoxaemic burden.

From a respiratory-medicine perspective, the most novel finding is the dissociation between the drivers of cardiovascular and pulmonary death. The Fine–Gray analyses reveal that the percentage of total sleep time spent below 90% oxygen saturation (Des < 90% TST), rather than CPAP-related variables, is the principal driver of cause-specific pulmonary mortality after competing risks are accounted for—emphasising the importance of hypoxaemic burden as a treatment target independent of the AHI. Nocturnal desaturation was the dominant predictor of pulmonary mortality in both populations, and retained independent prognostic importance in the full cohort after multivariable adjustment. The elevated subdistribution hazards associated with CPAP use in univariable Fine–Gray models reflect confounding by indication rather than a true adverse effect. The CPAP response classification introduced here extends the binary treatment paradigm by showing that the quality of AHI reduction on home CPAP therapy independently predicts survival; the dose–response gradient observed in the Kaplan–Meier curves (good responders > poor responders > no CPAP) corroborates observational findings of incremental mortality reduction with greater nightly usage [9,10,11]. Caution is nonetheless warranted, as the response classification is based on the mean residual AHI recorded by the device across therapy nights, and longitudinal adherence data were not available.

The apparent divergence between the standard and competing-risks analyses reflects the different questions these models address rather than an inconsistency in the data. The cause-specific Cox model estimates the instantaneous rate of cardiovascular death among patients still at risk and indicates a protective association of CPAP, whereas the Fine–Gray subdistribution models estimate effects on the cumulative incidence of cardiovascular death while retaining patients who die of competing causes—chiefly neoplasms and respiratory disease—in the risk set. Under a high competing-risk burden, a reduction in the cause-specific hazard need not translate into a lower cumulative incidence, and stratifying CPAP by response category further reduces group sizes and widens confidence intervals. The cause-specific and subdistribution hazards should therefore be regarded as complementary, and the cardiovascular survival advantage interpreted with corresponding caution.

Our findings contribute to resolving the well-documented discordance between observational studies and RCTs on CPAP and mortality [16]. The null findings of the SAVE trial [7] and negative meta-analyses of RCTs [17,18] may be explained by several factors: CPAP adherence in our cohort was defined as ≥4 h/night on ≥75% of nights—substantially exceeding the 3.3-h mean in SAVE [7]; our 8–20 year follow-up enabled detection of benefits that may require years of cumulative therapy to emerge [12,13]; and stratified analyses reveal that the benefit is confined to severe OSA, a subgroup effect inevitably diluted in intention-to-treat analyses of mixed-severity populations [8,14]. Regarding cardiovascular mortality specifically, a meta-analysis of nine studies reported a 66% reduction (HR 0.34, 95% CI 0.17–0.68) attributable predominantly to observational data [19], consistent with our finding of a 29% cardiovascular mortality reduction in the AHI ≥ 15 subgroup.

These findings carry direct implications for clinical practice and population respiratory health. First, they support concentrating CPAP resources and adherence-support efforts on patients with severe OSA, in whom the absolute survival benefit is greatest, rather than distributing them uniformly across all OSA severities. Second, the prognostic value of response quality argues for routine monitoring of device-recorded residual AHI as a modifiable treatment target, shifting the clinical goal from merely initiating CPAP to verifying that effective disease control is achieved during home use. Third, the divergent drivers of cardiovascular and pulmonary mortality—residual AHI control versus nocturnal hypoxaemic burden—suggest that oximetric endpoints deserve explicit attention alongside the AHI when titrating therapy and designing trials. Because baseline polysomnographic and clinical features predicted response category with high accuracy, such stratification could in principle be applied at the point of diagnosis, enabling more efficient enrichment of future randomised trials and more personalised management pathways. Finally, because the survival benefit of CPAP is concentrated in patients who achieve effective disease control, those with poor adherence or an inadequate therapeutic response warrant complementary, interdisciplinary management: early screening for OSA in dental settings and the use of mandibular advancement devices as an alternative for patients with low CPAP compliance may broaden effective treatment and improve outcomes [20].

Several limitations warrant consideration. The observational design precludes causal inference, and residual confounding—including the “healthy user” effect [8]—cannot be fully excluded. The CPAP response classification is based on device-recorded residual AHI averaged across therapy nights and does not capture longitudinal changes in adherence or efficacy. The classification models achieved macro F1-scores of 0.57–0.63, reflecting class imbalance—particularly for good responders—and require external validation before clinical deployment. Finally, the single-centre design may limit generalisability, and external validation of the prediction models in independent respiratory cohorts is required.

A further limitation is the potential selection bias inherent to the observational design: patients who received CPAP may have differed systematically from those who did not in ways only partly captured by the measured covariates. Despite adjustment for the available demographic, polysomnographic, and comorbidity variables, residual confounding by unmeasured factors (overall health status, socioeconomic position, motivation, adherence support) cannot be excluded, so the survival benefit should be interpreted as an association—possibly reflecting in part a “healthy user” effect—rather than proof of causation.

Because continued adherence throughout the 8–20-year follow-up could not be verified, and CPAP was modelled as a baseline exposure rather than a time-varying covariate, immortal-time bias and a healthy-user effect cannot be fully excluded; the observed association is best interpreted as reflecting treatment initiation rather than proven sustained protection.

Information on several potentially relevant comorbidities—chronic lung disease, heart failure, chronic kidney disease, and malignancy—was not collected in the database and could not be included as covariates, representing a further potential source of residual confounding.

## 5. Conclusions

Long-term CPAP therapy confers a significant, severity- and response-quality-dependent survival benefit in patients with moderate-to-severe OSA. The benefit is driven by the degree of residual AHI suppression achieved during routine home CPAP use, quantified as the mean residual AHI recorded by the device across all therapy nights. Nocturnal hypoxaemic burden, quantified as the absolute time spent with oxygen saturation below 90%, remained an independent predictor of cause-specific pulmonary mortality in both the full cohort and the AHI ≥ 15 subgroup after multivariable adjustment, while CPAP response category was independently associated with all-cause survival. The true beneficiaries of CPAP are therefore patients with severe disease who achieve effective control of residual events and nocturnal hypoxaemia. Future randomised trials should stratify by OSA severity and CPAP response quality, enforce rigorous adherence thresholds, and employ follow-up of sufficient duration to capture delayed mortality benefits [12,13].

## Figures and Tables

**Table 1:** Baseline demographic, clinical, polysomnographic, and treatment characteristics of the full study cohort (n = 4368). Continuous variables: median (IQR); categorical: n (%).

Variable	Category	Count/Value	Median (IQR)	Range
**Demographics**				
Gender	Male	3055 (69.94%)	–	–
	Female	1313 (30.06%)	–	–
Age (years)		n = 4368	58 (48.0–64.0)	18–93
BMI (kg/m^2^)		n = 4368	30.74 (27.44–34.77)	18.08–79.57
Neck circumference (cm)		n = 4368	42.5 (40.5–44.0)	29.8–60
**Polysomnographic Parameters and OSA Severity**				
AHI total (events/h)		n = 4368	16.5 (5.2–40.3)	0–148.7
AHI REM (events/h)		n = 4368	19.5 (4.3–45.5)	0–143.3
AHI NREM (events/h)		n = 4368	16.3 (3.9–41.9)	0–158.5
Desaturation < 90% (min)		n = 4368	9 (2.0–55.0)	0–554
Average saturation (%)		n = 4368	92 (90.7–94.0)	56–100
OSA severity	Severe	1450 (33.20%)	–	–
	Moderate	854 (19.55%)	–	–
	Mild	1006 (23.03%)	–	–
	Normal	1058 (24.22%)	–	–
**Comorbidities, Smoking, and Treatment**				
Hypertension		2617 (59.91%)	–	–
Diabetes mellitus		688 (15.75%)	–	–
Dyslipidemia		1016 (23.26%)	–	–
Atrial fibrillation		213 (4.88%)	–	–
Myocardial infarction		242 (5.54%)	–	–
Stroke		169 (3.87%)	–	–
Smoking	Never	1914 (43.82%)	–	–
	Former (≥10 years)	1311 (30.01%)	–	–
	Current	1143 (26.17%)	–	–
CPAP therapy	Yes	1193 (27.31%)	–	–
	No	3175 (72.69%)	–	–
CPAP therapy in AHI ≥ 15	Yes	956 (41.49% †)	–	–
CPAP therapy time (days)	n = 1052	169 (58–683)	15–4708	
	No	1348 (58.51% †)	–	–
CPAP response	Good	960 (21.98%)	–	–
	Poor	568 (13.00%)	–	–
	No CPAP	2840 (65.02%)	–	–
Residual AHI on CPAP (events/h)	Good	n = 960	3 (1.0–5.0)	–
	Poor	n = 568	16 (10.0–26.9)	–
AHI reduction from baseline (%)	Good	n = 960	93 (86.2–97.3)	–
	Poor	n = 568	42.2 (0.0–69.1)	–

† Percentage within the AHI ≥ 15 subgroup (n = 2304). CPAP therapy defined as ≥4 h per night on ≥75% of nights.

**Table 2:** Distribution of causes of death by ICD-10 system category in the full study cohort (n = 4368).

ICD-10 System Category	n (%)
Alive/not applicable	3351 (76.72%)
Cardiovascular diseases	335 (7.67%)
Neoplasms	243 (5.56%)
Respiratory diseases	101 (2.31%)
Symptoms and signs (not elsewhere classified)	98 (2.24%)
Digestive system diseases	49 (1.12%)
External causes	44 (1.01%)
Endocrine, nutritional, and metabolic diseases	35 (0.80%)
Special purposes (e.g., COVID-19)	31 (0.71%)
Other	81 (1.86%)

**Table 3:** Hazard ratios (95% CI) from multivariable Cox proportional hazards regression for all-cause, cardiovascular, and pulmonary mortality. Three models: (i) CPAP therapy (binary yes/no), (ii) AHI × CPAP interaction model, (iii) CPAP response classification (reference: No CPAP). All models adjusted for age, sex, BMI, AHI, oxygen desaturation, smoking, hypertension, diabetes, and hypothyroidism. Bold = *p* < 0.05. † CPAP therapy model.

	AHI ≥ 15 (n = 2304)		Full Cohort (n = 4368)	
Predictor	HR (95% CI)	*p*	HR (95% CI)	*p*
**All-Cause Mortality**				
CPAP therapy	0.75 (0.65–0.88)	† < 0.001	0.82 (0.71–0.94)	† 0.006
AHI (total)	2.14 (1.54–2.98)	<0.001	1.92 (1.49–2.48)	<0.001
AHI × CPAP	0.78 (0.65–0.94)	<0.01	0.90 (0.83–0.98)	<0.05
Desaturation < 90% (min)	1.06 (0.99–1.13)	0.096	1.07 (1.01–1.13)	<0.05
CPAP resp.: Poor	0.83 (0.68–1.01)	0.066	0.93 (0.77–1.12)	0.451
CPAP resp.: Good	0.66 (0.56–0.78)	<0.001	0.76 (0.65–0.88)	<0.001
**Cardiovascular Mortality**				
CPAP therapy	0.75 (0.58–0.97)	<0.05	0.85 (0.67–1.08)	0.186
AHI (total)	1.01 (0.99–1.03)	0.177	1.02 (1.00–1.03)	<0.05
AHI × CPAP	0.99 (0.99–1.00)	<0.05	0.99 (0.99–1.00)	<0.05
Desaturation < 90% (min)	1.00 (1.00–1.00)	<0.05	1.00 (1.00–1.00)	0.859
CPAP resp.: Poor	0.89 (0.64–1.25)	0.505	1.10 (0.81–1.49)	0.541
CPAP resp.: Good	0.73 (0.55–0.96)	0.026	0.80 (0.61–1.05)	0.106
**Pulmonary Mortality**				
CPAP therapy	0.54 (0.33–0.90)	<0.05	0.56 (0.35–0.89)	<0.05
AHI (total)	1.03 (1.00–1.06)	0.090	1.03 (1.00–1.05)	<0.05
AHI × CPAP	0.99 (0.98–1.00)	0.141	0.99 (0.98–1.00)	0.077
Desaturation < 90% (min)	1.00 (1.00–1.00)	<0.01	1.00 (1.00–1.00)	<0.001
CPAP resp.: Poor	0.61 (0.32–1.19)	0.147	0.65 (0.35–1.20)	0.168
CPAP resp.: Good	0.58 (0.34–0.99)	0.044	0.60 (0.37–0.99)	0.048

CPAP therapy: binary model (no interaction term). AHI × CPAP: interaction model. CPAP response: separate model replacing binary CPAP (reference: No CPAP).

**Table 4:** Hazard ratios (95% CI) from univariable cause-specific Cox proportional hazards regression for cardiovascular and pulmonary mortality in the AHI ≥ 15 subgroup (n = 2304) and the full cohort (n = 4368). Each predictor was modelled individually without adjustment. Bold = *p* < 0.05.

	AHI ≥ 15 (n = 2304)		Full Cohort (n = 4368)	
Predictor	HR (95% CI)	*p*	HR (95% CI)	*p*
**Cardiovascular Mortality**				
CPAP therapy	0.86 (0.67–1.10)	0.227	1.42 (1.13–1.78)	<0.01
AHI (total)	1.01 (1.00–1.01)	<0.01	1.02 (1.01–1.02)	<0.001
AHI CPAP	1.00 (0.99–1.00)	0.711	1.01 (1.00–1.01)	<0.001
Desaturation < 90% (min)	1.00 (1.00–1.00)	<0.05	1.00 (1.00–1.00)	<0.001
CPAP resp.: Poor	1.13 (0.82–1.57)	0.462	2.05 (1.54–2.73)	<0.001
CPAP resp.: Good	0.80 (0.61–1.06)	0.117	1.40 (1.09–1.81)	0.010
**Pulmonary Mortality**				
CPAP therapy	0.73 (0.45–1.18)	0.204	1.13 (0.73–1.75)	0.576
AHI (total)	1.01 (1.01–1.02)	<0.01	1.02 (1.01–1.02)	<0.001
AHI CPAP	1.00 (0.99–1.01)	0.782	1.01 (1.00–1.01)	0.086
Desaturation < 90% (min)	1.00 (1.00–1.00)	<0.001	1.00 (1.00–1.01)	<0.001
CPAP resp.: Poor	0.95 (0.51–1.79)	0.884	1.50 (0.85–2.63)	0.163
CPAP resp.: Good	0.70 (0.42–1.17)	0.169	1.17 (0.73–1.87)	0.522

CPAP therapy: binary model (yes vs. no). AHI CPAP: residual apnea–hypopnea index on CPAP. CPAP response categories (Good, Poor) are compared with the reference category (No CPAP).

**Table 5:** Selected continuous variables by CPAP response category. Values: median (IQR). *p*: Kruskal–Wallis. Post hoc column shows Bonferroni-corrected pairwise comparisons (Dunn’s test, adjusted *p* < 0.05); the three symbols correspond, in order, to: No CPAP vs. Poor, No CPAP vs. Good, Poor vs. Good. * = significant; – = not significant.

Variable	No CPAP	Poor	Good	*p*	Post Hoc
AHI total	9.2 (3.1–23.0)	39.8 (12.4–67.1)	36.8 (21.7–59.4)	<0.001	*, *, –
AHI NREM	10.6 (2.6–29.0)	28.4 (8.6–62.2)	31.6 (16.0–56.5)	<0.001	*, *, –
AHI REM	13.0 (2.7–33.3)	32.3 (7.5–60.6)	36.2 (15.0–59.5)	<0.001	*, *, –
Desaturation < 90% (min)	7.00 (1.00–35.85)	16.95 (4.00–96.93)	27.00 (6.00–92.53)	<0.001	*, *, –
Avg. saturation	93.0 (91.0–94.0)	91.1 (88.0–93.0)	92.0 (90.0–93.0)	<0.001	*, *, *
Age (years)	57.0 (46.0–64.0)	58.0 (52.0–64.0)	59.0 (50.0–65.0)	<0.001	*, *, –
BMI	29.9 (26.8–33.6)	33.6 (29.2–38.3)	32.0 (29.0–36.6)	<0.001	*, *, *
Neck circ. (cm)	42.0 (40.0–44.0)	43.5 (42.0–46.0)	43.0 (41.0–45.0)	<0.001	*, *, *
Epworth	8.0 (4.0–11.0)	9.0 (6.0–13.0)	9.0 (6.0–12.0)	<0.001	*, *, –
Back AHI index	19.2 (6.0–54.6)	61.0 (22.0–91.9)	69.7 (37.9–107.3)	<0.001	*, *, *
Sys. BP (mmHg)	130 (120–145)	135 (120–145)	135 (125–150)	<0.001	–, *, –
Dias. BP (mmHg)	85 (80–90)	85 (80–95)	85 (80–95)	<0.001	*, *, –
Residual AHI on CPAP (AHI_CPAP)	not applicable	16 (10–26.9)	3 (1–5)	<0.001	–, –, *
AHI reduction from baseline (%)	not applicable	42.2 (0–69.1)	93 (86.2–97.3)	<0.001	–, –, *

**Table 6:** Distribution of categorical variables by CPAP response category. Values: n (% within category). *p*: Chi-squared test.

Variable	No CPAP	Poor	Good	*p*
**Patient Characteristics**				
Gender—Male	1902 (62.3%)	444 (14.5%)	709 (23.2%)	<0.001
**Comorbidities**				
Hypertension	1550 (59.2%)	404 (15.4%)	663 (25.3%)	<0.001
Diabetes	357 (51.9%)	132 (19.2%)	199 (28.9%)	<0.001
Dyslipidemia	585 (57.6%)	175 (17.2%)	256 (25.2%)	<0.001
Myocardial infarction	140 (57.9%)	43 (17.8%)	59 (24.4%)	0.028
Atrial fibrillation	122 (57.3%)	43 (20.2%)	48 (22.5%)	0.004

## Data Availability

The de-identified data underlying this article cannot be shared publicly because they contain sensitive clinical and national death-registry information governed by institutional and national data-protection regulations. De-identified data may be made available by the corresponding author upon reasonable request and subject to approval by the Bioethics Committee of the Medical University of Łódź and execution of an appropriate data-use agreement. The analysis code is available from the corresponding author on reasonable request.

## Supplementary Materials

The following supporting information can be downloaded at: https://www.mdpi.com/article/10.3390/arm94040055/s1, File S1. STROBE Statement—Checklist for Cohort Studies; Figure S1: The confusion matrix—logistic regression: no CPAP, poor and good CPAP adherence to predicted no CPAP, poor and good CPAP adherence. Reference [21] is cited in Supplementary Materials.

## Abbreviations

The following abbreviations are used in this manuscript:

OSA	Obstructive sleep apnea
CPAP	Continuous positive airway pressure
AHI	Apnea–hypopnea index
PSG	Polysomnography
TST	Total sleep time
SpO_2_	Peripheral oxygen saturation
HR	Hazard ratio
CI	Confidence interval
CIF	Cumulative incidence function
RSF	Random survival forest
RF	Random forest
AUC	Area under the receiver operating characteristic curve
ESS	Epworth Sleepiness Scale
ICD-10	International Classification of Diseases, 10th Revision
BMI	Body mass index
IBS	Integrated Brier Score
