# Supplementary material for: Who Really Benefits from CPAP? Disease Severity, Response Quality, and Long-Term Survival in Obstructive Sleep Apnea
Source: Adv Respir Med. 2026 Jul 28;94(4):55. doi: 10.3390/arm94040055 (PMC13509334; doi:10.3390/arm94040055)

File S1. STROBE Statement—Checklist for Cohort Studies

Manuscript: Who Really Benefits from CPAP? Disease Severity, Response Quality, and Long-Term Survival in Obstructive Sleep Apnea

Journal: *Advances in Respiratory Medicine*

Item No	Recommendation	Location / How addressed
**Title and abstract**		
**1**	(a) Indicate the study's design with a commonly used term in the title or the abstract. (b) Provide an informative and balanced summary of what was done and what was found.	Title; Abstract (structured single paragraph: Background, Methods, Results, Conclusions). Cohort design and machine-learning approach stated in the title and abstract.
**Introduction**		
**2**	Explain the scientific background and rationale for the investigation being reported.	Introduction (Section 1), paragraphs 1–4 (OSA–mortality association; observational–RCT discordance).
**3**	State specific objectives, including any prespecified hypotheses.	Introduction (Section 1), final paragraph (explicit hypothesis on response quality; three numbered aims).
**Methods**		
**4**	Present key elements of study design early in the paper.	Section 2.1, Study Population and Data Source (retrospective single-centre cohort).
**5**	Describe the setting, locations, and relevant dates, including periods of recruitment, exposure, follow-up, and data collection.	Section 2.1 (tertiary sleep centre, Medical University of Łódź; 8–20-year follow-up; national death-registry linkage).
**6**	(a) Give the eligibility criteria, and the sources and methods of selection of participants. Describe methods of follow-up. (b) For matched studies, give matching criteria and number of exposed/unexposed.	Section 2.1 (consecutive PSG referrals; exclusions: age/BMI < 18, follow-up criteria, missing data). Not a matched study.
**7**	Clearly define all outcomes, exposures, predictors, potential confounders, and effect modifiers. Give diagnostic criteria, if applicable.	Sections 2.1 and 2.6 (OSA severity; CPAP therapy definition; CPAP response classification; ICD-10 outcome ascertainment). Confounders listed in the adjustment sets (Sections 2.3–2.5).
**8**	For each variable of interest, give sources of data and details of methods of assessment (measurement). Describe comparability of assessment methods if there is more than one group.	Sections 2.1 and 2.6 (PSG-derived variables; device-recorded residual AHI; registry-based mortality; comorbidities and medications from clinical records).
**9**	Describe any efforts to address potential sources of bias.	Sections 2.3–2.5 (multivariable adjustment; Fine–Gray competing-risks framework); Discussion, Section 4 (confounding by indication and "healthy user" effect addressed).
**10**	Explain how the study size was arrived at.	Section 2.1 (all eligible consecutive patients; full cohort n = 4,368; prespecified AHI ≥ 15 subgroup n = 2,304).
**11**	Explain how quantitative variables were handled in the analyses. If applicable, describe which groupings were chosen and why.	Sections 2.1 and 2.6 (OSA severity categories; CPAP response categories Good/Poor/No CPAP; continuous predictors modelled per unit with the PH assumption checked in Section 2.3).
**12**	(a) Describe all statistical methods, including those used to control for confounding. (b) Describe methods used to examine subgroups and interactions. (c) Explain how missing data were addressed. (d) If applicable, explain how loss to follow-up was addressed. (e) Describe any sensitivity analyses.	Sections 2.3–2.8 (Cox PH, cause-specific Cox, Fine–Gray; AHI × CPAP interaction; AHI ≥ 15 subgroup; patients with missing key variables excluded; no loss to follow-up for vital status; bootstrap CIF comparisons; ML — Random Forest, multinomial logistic regression).
**Results**		
**13**	(a) Report numbers of individuals at each stage. (b) Give reasons for non-participation at each stage. (c) Consider use of a flow diagram.	Section 3.1 and Table 1 (consecutive referrals → eligible cohort → deaths/censored → analysis subgroups).
**14**	(a) Give characteristics of study participants and information on exposures and potential confounders. (b) Indicate number of participants with missing data for each variable of interest. (c) Summarise follow-up time.	Section 3 — Table 1 (baseline characteristics); Table 2 (cause-of-death distribution); follow-up 8–20 years.
**15**	Report numbers of outcome events or summary measures over time.	Section 3 / Table 2 (1,017 deaths; 335 cardiovascular, 101 respiratory); AHI ≥ 15 subgroup 737 deaths.

Item No	Recommendation	Location / How addressed
**16**	(a) Give unadjusted and confounder-adjusted estimates and their precision (95% CI). (b) Report category boundaries when continuous variables were categorized. (c) If relevant, translate estimates of relative risk into absolute risk.	Sections 3.1–3.2 — Tables 3–4 (adjusted and univariable HRs with 95% CI); Figures 1–4 (KM and CIF); category boundaries defined in Section 2.6.
**17**	Report other analyses done—e.g. analyses of subgroups and interactions, and sensitivity analyses.	Sections 3.1–3.4 — AHI × CPAP interaction; severity-stratified KM; CPAP-response-stratified analyses; ML prediction (Figure 5, Tables 5–6).
**Discussion**		
**18**	Summarise key results with reference to study objectives.	Discussion (Section 4), paragraph 1 (severity- and response-dependent CPAP benefit).
**19**	Discuss limitations of the study, taking into account sources of potential bias or imprecision. Discuss both direction and magnitude of any potential bias.	Discussion (Section 4), limitations paragraph (observational design, residual confounding, response-classification limits, model class imbalance).
**20**	Give a cautious overall interpretation of results considering objectives, limitations, multiplicity of analyses, results from similar studies, and other relevant evidence.	Discussion (Section 4) — reconciliation with SAVE/RCTs and meta-analyses; clinical and public-health implications paragraph.
**21**	Discuss the generalisability (external validity) of the study results.	Discussion (Section 4) — single-centre cohort; external validation of prediction models flagged as required.
**Other information**		
**22**	Give the source of funding and the role of the funders for the present study and, if applicable, for the original study on which the present article is based.	Back matter — Funding statement and Institutional Review Board Statement (no specific external funding; educational programme had no role).

Note: Section numbers refer to the numbered sections of the submitted manuscript. An explanation and elaboration article discusses each checklist item and is available at www.strobe-statement.org [21].

Figure S1. *The confusion matrix – logistic regression: no CPAP, poor and good CPAP adherence to predicted no CPAP, poor and good PAP adherence.*

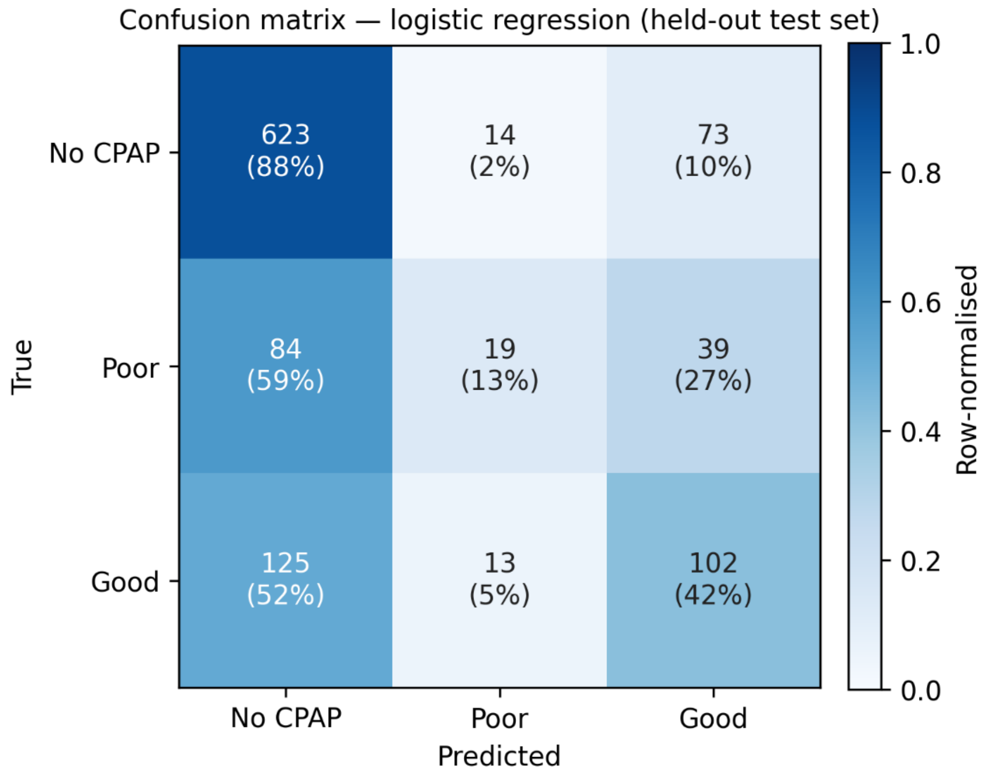

Supplement: Supplementary file 1 [file arm-94-00055-s001.zip › arm-4428996-supplementary.pdf]
